# Fluid Overload and Graft Injury Following Pediatric Liver Transplantation: A Single-Center Analysis

**DOI:** 10.3390/jcm14113759

**Published:** 2025-05-27

**Authors:** Sapir Bar, Yael Mozer Glassberg, Michael Gurevich, Elhanan Nahum, Avichai Weissbach, Eytan Kaplan, Gili Kadmon

**Affiliations:** 1 Pediatric Intensive Care Unit, Schneider Children’s Medical Center in Israel, 14 Kaplan Street, Petach Tikva 4920235, Israel; sapirbar5@gmail.com (S.B.); nahum@clalit.org.il (E.N.); avihaiw@clalit.org.il (A.W.); eytank@clalit.org.il (E.K.); 2 Gastroenterology Institute, Schneider Children’s Medical Center in Israel, Petach Tikva 4920235, Israel; yaelm3@clalit.org.il; 3 Faculty of Medicine, Tel Aviv University, Tel Aviv 6997801, Israel; mgurevich@clalit.org.il; 4 Pediatric Transplantation and Liver Surgery Unit, Schneider Children’s Medical Center in Israel, Petach Tikva 4920235, Israel

**Keywords:** children, complications, fluid balance, hepatology, treatment, transaminases, vascular injury

## Abstract

**Background:** We aimed to compare graft injury and complications after liver transplantation in children with higher versus lower fluid balance. **Methods:** In a cohort of 79 pediatric liver transplant recipients, we analyzed the associations of decreases in alanine aminotransferase (ALT) and bilirubin (delta ALT, delta bilirubin) with fluid balance in the first six postoperative days and associations of fluid balance with vascular complications and mean ALT one year after the transplantation. **Results:** Patients who developed vascular complications had significantly higher mean cumulative fluid balance during the first three postoperative days, as well as higher mean fluid balance on postoperative days 0 (POD0) and 2 (POD2) (*p* < 0.05), compared to those without complications. A negative correlation was observed between fluid balance and delta ALT on POD2. Additionally, patients with a cumulative fluid balance exceeding 200 mL/kg during the first three postoperative days had higher mean ALT levels one year after transplantation (*p* = 0.03). **Conclusions:** Fluid overload was associated with vascular complications and showed correlations with markers of graft injury. Prospective studies are needed to validate these findings and further clarify the role of fluid balance in pediatric liver transplantation.

## 1. Introduction

During the last few decades, pediatric liver transplantation rates have significantly increased [1]. This is due to broader indications for liver transplantation in children, including liver tumors and metabolic disorders and to the expanding pool of available organs and the higher success rates, as reflected by 5-year survival of over 85% [2,3,4,5,6]. Nevertheless, intraoperative and postoperative complications of liver transplantation in children are still common, including significant fluid and blood losses, hemodynamic instability, and vascular complications [3,5,7,8,9]. Therefore, fluid administration and ionotropic support are the mainstay of treatment during and following transplantation. Fluid under-resuscitation might cause low blood pressure, impaired graft perfusion, and increased risk of vascular thrombosis and kidney injury. However, fluid overload might expose patients to other complications, including pleural effusions, ascites, and liver edema [10,11].

Several studies in adults have demonstrated correlations between fluid overload and adverse outcomes following liver transplantation, specifically early graft dysfunction and mortality [11,12,13]. Among critically ill children in general, fluid overload has been associated with worse outcomes, including impaired respiratory function, acute kidney injury, prolonged intensive care stay, and increased mortality [14,15,16]. However, in the specific population of pediatric liver transplant recipients, the associations between fluid overload, complications, and graft dysfunction remain inconclusive.

In this single-center, retrospective study, we aimed to evaluate the correlation between fluid balance and graft injury during the immediate postoperative period and 1 year after pediatric liver transplantation.

## 2. Patients and Methods

### 2.1. Study Design and Settings

A retrospective chart review study was conducted at Schneider Children’s Medical Center of Israel. This 300-bed, tertiary, university-affiliated pediatric hospital is the only pediatric liver transplantation center in Israel. The study was approved by the institutional review board, and the need for informed consent was waived by the board.

### 2.2. Study Population

The study group consisted of all patients who underwent liver transplantation, subsequently hospitalized at the pediatric intensive care unit (PICU) during 2010–2021, and who underwent clinical and laboratory follow-up at the gastroenterology clinic until 2022. Three patients with significant vascular complications (i.e., multiple revisions of the hepatic artery) during the surgery were excluded. This is due to the intention to evaluate the effect of fluid therapy on graft function and to limit the effect of graft ischemic time.

### 2.3. Liver Transplantation Management

According to the protocol followed in our department throughout the study period, fluids and inotropes, usually dopamine, were administered during surgery, as required to maintain normal blood pressure. At the end of the transplantation, the patients were immediately transferred to the PICU from the operation theater while they were mechanically ventilated. At the PICU, they were treated with maintenance fluids containing saline with 5–10% glucose, according to blood glucose levels, and another saline solution with 2.5% albumin as a 1:1 replacement for abdominal drain secretions. Blood pressure was monitored with an arterial catheter, and central venous pressure (CVP) was monitored with an internal jugular vein catheter. We aimed to maintain systolic blood pressure (SBP) above the 50th percentile for age, and dopamine, adrenaline, and noradrenaline infusions were administered as required. Upon arrival and frequently thereafter, the fluid status of patients was evaluated by parameters of heart rate, blood pressure, pulse pressure variation, peripheral perfusion, urine output, and CVP. If hypovolemia was suspected by the treating physician, a fluid bolus of a saline solution was administered. Conversely, if fluid overload was noted, the fluid administration rate was reduced, or a furosemide infusion was initiated. Blood products were given according to the following criteria: red blood cells for hemoglobin levels below 7 g/dL, fresh frozen plasma for INR values greater than 5, and platelet transfusions for counts below 20,000/μL. In cases of active bleeding, transfusion thresholds were modified, aiming for a hemoglobin level above 8 g/dL, platelet counts over 100,000/μL, and near-normal coagulation parameters. Repeated blood tests were taken for blood count, blood biochemistry, ammonia, coagulation indices, and blood gases. Analgesia and sedation were provided by midazolam, morphine, and dexmedetomidine infusions. Typically, heparin infusion commenced several hours post-transplantation, in the absence of signs of significant abdominal bleeding. The infusion was replaced by enoxaparin 2–3 days after the surgery. In addition, antiaggregation treatment with aspirin was introduced once platelet levels surpassed 100,000 and feeding was resumed. In the event of a vascular complication (i.e., stenosis or thrombosis of hepatic artery, hepatic vein, or portal vein), heparin infusion was increased to a therapeutic dose, and particular attention was given to maintaining intravascular volume to prevent hypovolemia. Routine liver Doppler ultrasound examinations were performed in the operating room at the end of the transplantation, upon admission to the PICU, and each morning for 5 to 7 days post-transplant. In cases of vascular complications, these examinations were continued for an extended duration. Tracheal extubation was usually performed on postoperative day 1 (POD1).

During the study period, we followed a constant immunosuppression protocol, including induction with basiliximab. The maintenance protocol consisted of a tacrolimus-based drug regimen and steroids. Tacrolimus dosages were adjusted to achieve trough levels at 8–12 ng/mL. Methylprednisolone was started intravenously at a dose of 10 mg/kg on POD0, reduced to 5 mg/kg on POD1, and then reduced by 1 mg/kg each day, down to 1 mg/kg on POD5 and 0.5 mg/kg from POD6 onward.

### 2.4. Data Collection

Data were collected from the medical digital records of the operation and of the PICU hospitalization (Metavision^®^, iMDsoft, Tel Aviv, Israel). The medical record information included epidemiological data, the type of donor (living-related vs. deceased donation), ischemic time, graft size, surgery duration, vital signs, fluid balance, and treatments administered during the transplantation. Blood test results were documented and included alanine aminotransferase (ALT), aspartate aminotransferase, gamma glutamyl transpeptidase, bilirubin, creatinine, lactate, and ammonia levels. These levels were assessed at baseline (immediately before the transplantation), at the time of the ALT peak value (usually at PICU admission or up to several hours after the operation), and at 7 a.m. on POD1-5. In addition, fluid balance, CVP, and arterial catheter systolic blood pressures during the operation and at 7 a.m. on POD0-5 were collected. The vaso-inotropic score (VIS) is a weighted sum of all administered inotropes and vasoconstrictors. This score, which reflects pharmacological support of the cardiovascular system [17], was calculated during the surgery and at 7 a.m. on POD0-5. Data were also collected of daily liver ultrasound Doppler and chest radiography findings, including pulmonary congestion, as interpreted by a pediatric radiologist, and of complications during the operation and PICU hospitalization. Surgical complications included hypotension episodes with SBP below the 5th percentile per age, major bleeding, and revisions of the hepatic artery or the portal or hepatic vein. Complications during PICU hospitalization included hemorrhage, vascular complications, acute rejection, and infections. The length of invasive and noninvasive mechanical ventilation, the length of PICU hospitalization, and liver enzyme and bilirubin values one year following the transplantation were collected as well.

### 2.5. Definitions

Daily fluid balance was defined as the difference between daily fluid intake (e.g., medications, fluid infusion, blood products excluding packed red blood cells, oral intake) and daily fluid loss (e.g., urine output, nasogastric tube, and abdominal drain secretions). Fluid balance/kg = net fluid balance/body weight.

Delta ALT and delta bilirubin were defined as the proportions of change in ALT and bilirubin levels between consecutive postoperative days. For example, delta ALT on POD1 = (ALT peak value − ALT POD1) × 100/ALT peak value. Delta bilirubin on POD2 = (bilirubin POD1-bilirubin POD2) × 100/bilirubin POD1 (a positive value indicates improvement in liver function).

SBP percentiles—for each patient, SBP values were converted to SBP percentiles by age and height, according to the clinical practice guideline for high blood pressure in children [18].

Severe AKI was defined as a serum creatinine level greater than twice the baseline value, in accordance with the Kidney Disease Improving Global Outcomes (KDIGO) criteria for stage 2 or 3 AKI.

### 2.6. Statistical Analysis

Data were analyzed with an Excel spreadsheet (Microsoft Corp., Redmond, WA, USA). Patient characteristics are presented by medians and interquartile ranges (IQR). To identify factors that contributed to improved liver function following transplantation, associations were analyzed of delta ALT and delta bilirubin with the following parameters: fluid balance/kg, CVP, SBP percentile, abnormal ultrasound Doppler examination, and the presence of pulmonary congestion on chest radiograph on every postoperative day. Associations were analyzed of the accumulated delta ALT of POD0-3 with patient age, weight, height, gender, baseline diagnosis, donor type (living vs. cadaveric), graft type (whole vs. split), accumulated fluid balance of POD0-3, and intraoperative parameters. The latter included CVP, VIS, and hypotension episode durations. Continuous parameters were analyzed using Pearson correlation and categorical parameters with analysis of variance (ANOVA). Levene’s test for variance was performed, and, when the result was statistically significant, a Welch equality of means test was performed. In addition, the *t*-test was used to assess the difference in mean fluid balance between patients with and without vascular complications, and the Fisher exact test was used to compare differences in the incidence of severe AKI between patients with higher versus lower cumulative fluid balance on postoperative days 0–3. Finally, the Mann–Whitney U test was used to compare the difference in mean ALT at 1 year after the transplantation and between patients with higher and lower fluid balance during the first 3 postoperative days. Twenty patients with significant complications at 1 year (i.e., acute rejection, significant vascular or biliary complications) were excluded from this analysis. A *p* < 0.05 was considered significant.

## 3. Results

### 3.1. Patient Characteristics

Seventy-nine liver transplant patients were included. Of the patients, 41 transplantations were living-related, and 38 were deceased donations (25 whole and 14 split liver). The median ischemic time, available for only 16 patients, was 5.5 (range 0.5–12) hours. The demographic and clinical characteristics of the patients are summarized in Table 1. Data were available for all patients during the first three postoperative days. On postoperative days 4 and 5, data were available for 77 (97%) and 70 (89%) patients, respectively, due to earlier PICU discharge in some cases.

### 3.2. Complications

The median (IQR) duration of the transplantations was 548 (440–651) minutes. During the transplantation, hypotension occurred in 51 (65%) patients. The median (IQR) duration of the hypotensive episodes was 28 (12.5–47) minutes. Sixty-seven (85%) patients required blood products transfusion; seventy-one (90%) patients were treated with inotropes during the surgery, mainly dopamine. The median (IQR) VIS was 7.5 (4.5–10).

Following the transplantation, 51 episodes of complications occurred (Table 2). The most common types of infection were peritonitis, pneumonia, and bacteremia. A total of nine episodes of vascular complications occurred: hepatic vein stenosis in four, hepatic artery thrombosis in four, and portal vein thrombosis in one patient. All the patients were treated with anticoagulation; four patients underwent surgical revision (hepatic artery in three and portal vein in one), and two patients underwent stent insertion into the inferior vena cava. Other complications included nine episodes of biliary leak, requiring surgical revision; eight episodes of postoperative bleeding, mainly abdominal and requiring surgical revision in seven patients; and two episodes of tacrolimus poisoning. The latter manifested as encephalopathy in one patient and confusion in the other and resolved after transiently stopping tacrolimus treatment. One patient died (1.2%) at 5 months after the transplantation, following hemophagocytosis, bone marrow transplantation, and cytomegalovirus and aspergillus infection.

### 3.3. Fluid Balance

The median (IQR) fluid balance/kg was 187 mL/kg (121–260) at the end of the transplantation and the median (IQR) accumulated fluid balance of the first 3 postoperative days was 237 mL/kg (160–318). Pulmonary congestion was observed in 35 (44%) patients on POD0 and 27 (34%) patients on POD3. Among patients with an accumulated fluid balance exceeding 200 mL/kg, the median duration of noninvasive mechanical ventilation was longer than that of patients with a lower balance (2.6 vs. 1 day, *p* = 0.03). The median durations of invasive mechanical ventilation and of PICU hospitalization did not differ between the two groups.

**Figure 1:**
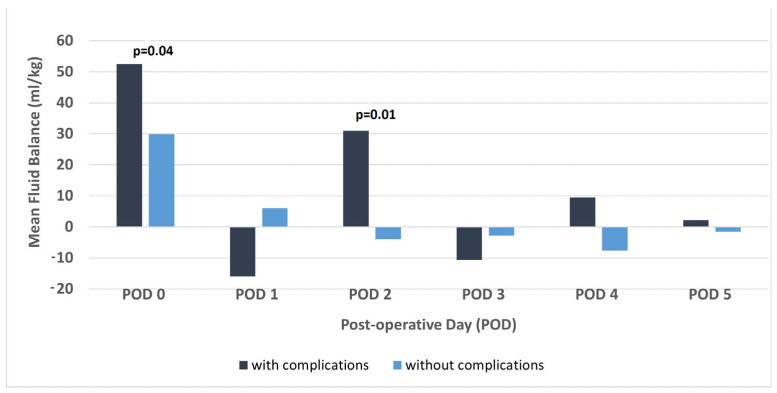
Daily fluid balance in patients with and without vascular complications. The mean fluid balance is shown for postoperative days 0–5, for patients with and without vascular complications.

Compared to patients without vascular complications, among those with complications on POD5, the mean values of daily fluid balance on POD0 and POD2 were higher (Figure 1, *p* = 0.04 and *p* = 0.01, respectively) and the mean accumulated fluid balance of the first 3 postoperative days was higher (347 vs. 243 mL/kg, *p* = 0.04). No correlations were found between vascular complications and CVP or SBP percentiles. No differences were found in the clinical characteristics of patients with and without vascular complications, including age, weight, underlying diagnosis, and type of transplantation.

### 3.4. Hemodynamics

The patients were kept hypertensive during their PICU hospitalization. Median (IQR) SBP percentiles were 99 (33–99) on POD0 and 99 (92–99) on POD5. The median (range) CVP was 8 (5–18) mmHg on POD0, 9 (5–16) mmHg on POD1, 7 (3–16) mmHg on POD2, and 6 (3–14) mmHg on POD3.

### 3.5. Postoperative Graft Function

Liver enzyme values increased following transplantation; they reached maximum values during the first postoperative hours and then gradually decreased. The mean (SD) maximum level of ALT was 618 (737) iu/L, and the mean (SD) maximum level of bilirubin was 7.2 (10.4) mg/dL.

Pearson correlation demonstrated a weak negative correlation between delta ALT and the daily fluid balance on POD2 (R = −0.36, *p* = 0.001). The same trend was evident on POD4 and POD5, though without statistical significance. On the contrary, Pearson correlation demonstrated weak positive correlations between delta bilirubin and the daily fluid balance on POD1 and POD4 but not on the other postoperative days.

No correlations were found of delta ALT or delta bilirubin with CVP, pulmonary edema, or SBP percentiles, nor were correlations found of the accumulated delta ALT of POD0-3, with any of the following parameters: patient gender, age, height, body weight, baseline diagnosis, donor and graft type (living vs. cadaveric), and clinical parameters recorded during the transplantation. The latter included CVP, VIS, and the duration of hypotension episodes.

### 3.6. Acute Kidney Injury

Prior to transplantation, six patients (8%) had pre-existing kidney disease, including one patient with end-stage renal disease undergoing hemodialysis. Severe AKI occurred in 22 patients (28%) during postoperative days 0–3. The incidence of severe AKI was not significantly different between patients with a cumulative fluid balance ≥ 200 mL/kg and those with <200 mL/kg (36% vs. 26%, *p* = 0.45).

### 3.7. Long-Term Graft Function

The median (IQR) durations to normalization of AST, ALT, and bilirubin were 6 (4–13), 11 (7–23), and 8 (2–17) days after the transplantation, respectively. No correlations were found of fluid balance with the time to liver enzyme or bilirubin normalization.

**Figure 2:**
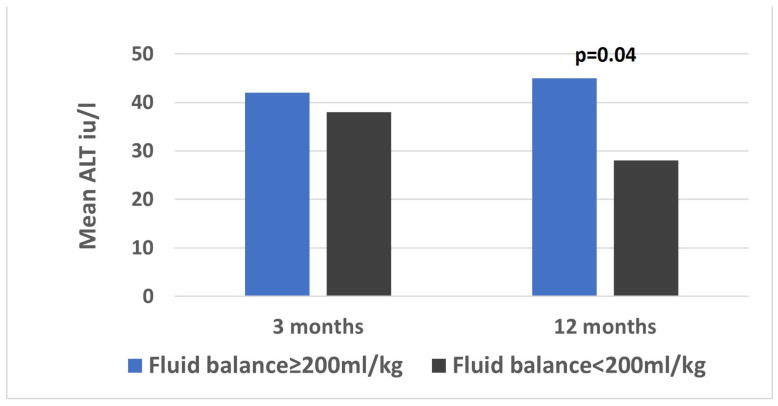
Long-term ALT in patients with high and low fluid balance. Mean values are shown of alanine aminotransferase (ALT) at 3- and 12-months postoperative among patients with accumulated fluid balance ≥ 200 and <200 mL/kg.

One year after the transplantation, data were available for 76 (96%) patients. The median ALT was 28 (17–40) iu/L and the median bilirubin (IQR) was 0.4 (0.3–0.6) mg/dL. The mean ALT was higher among patients with an accumulated fluid balance of 200 mL/kg or more, compared to those with a balance below 200 mL/kg on the first 3 postoperative days (44 vs. 25 iu/L, *p* = 0.04, Figure 2).

## 4. Discussion

In this retrospective, single-center analysis of 79 pediatric patients after liver transplant, we found several correlations between fluid overload and indices of graft injury. First, among patients with vascular complications compared to those without, the accumulated fluid balance of the first 3 postoperative days and the mean fluid balance on POD0 and 2 were higher. Second, delta ALT (i.e., the decrease in ALT values) was significantly higher among patients with lower than higher fluid balance on POD2. The same trend was evident on POD4 and POD5, though without statistical significance. Lastly, one year after the transplantation, the mean ALT was higher among patients with higher accumulated fluid balance on the first 3 postoperative days than among those with lower fluid balance.

Our demonstration of correlations between fluid overload and graft injury concurs with several studies in adults, which demonstrated associations of fluid overload during liver transplantation or postoperative weight gain, with early graft dysfunction [11,12,13].

In addition to its possible effect on graft injury, fluid overload also impaired pulmonary functions. Among patients with a fluid balance exceeding 200 mL/kg during the first 3 days, compared to patients with a lower fluid balance, the median duration of non-invasive mechanical ventilation was longer. Importantly, the need for prolonged noninvasive ventilation did not extend the PICU length of stay. This reflects our institutional practice, where children on noninvasive ventilation may be transferred to a general pediatric ward.

This finding of the prolonged need for respiratory support is consistent with two previous studies in pediatric liver transplant recipients. One study reported that high-volume fluid administration during transplantation was associated with increased blood loss, pulmonary edema, and prolonged hospital stay [19]. Another study found that a higher postoperative fluid balance (>20% of body weight) correlated with worse outcomes, including longer PICU and hospital stays, as well as fewer ventilator-free days [20]. In contrast, a study of pediatric renal transplant patients found no association between fluid overload and duration of respiratory support [21]. This difference may be explained by the increased susceptibility of liver transplant recipients to fluid overload–related respiratory failure, possibly due to their younger age and significant postoperative abdominal distension [20,22].

Our study demonstrates an association between fluid overload and vascular complications. Patients who developed vascular complications had a higher mean fluid balance on POD0 and 2, as well as a higher cumulative fluid balance over the first three postoperative days. However, no significant correlations were found between vascular complications and either CVP or SBP percentiles. This may be due to the fact that CVP was influenced by multiple factors beyond fluid status, such as abdominal distension, congenital cardiac defects, and impaired cardiac function. Additionally, we deliberately maintained higher SBP percentiles in this population to support graft perfusion, which may have contributed to the absence of detectable differences between the groups. However, it is also possible that fluid overload was a consequence rather than a cause of vascular complications; in these patients, fluid balance may have been kept intentionally high to prevent dehydration, which could potentially elevate the risk of thrombosis.

Kidney injury is a well-recognized complication following liver transplantation. In our cohort, 28% of patients developed severe AKI, defined as KDIGO stage 2 or 3. This incidence is comparable to that reported by Winters et al. [20] in their study examining fluid balance in pediatric liver transplantation. The study demonstrated an increased risk of severe AKI among patients with a fluid balance of 10–20% and >20% of body weight. In contrast, our analysis did not reveal a significant difference in the incidence of severe AKI between patients with a cumulative fluid balance > 200 mL/kg during POD0–3 (equivalent to >20% of body weight) and those with lower fluid accumulation. This discrepancy may be attributed to methodological differences, as our fluid balance calculations included intraoperative fluid administration, which was not accounted for in the study by Winters et al. [20]. Alternatively, the lack of a significant difference may reflect the smaller sample size of our cohort, potentially reducing the statistical power to detect such an association.

In this study, we evaluated bilirubin and ALT as markers of graft function and injury [13,23]. Other markers of graft function, such as INR, PT, and factor 5 levels were not reliable enough, as about half of our patients were treated with fresh frozen plasma during the transplantation or the first 3 subsequent days. Lactate, another marker of graft function, is also a marker of hemodynamic status and is often increased during adrenaline infusion [24]. As many of our patients received the latter, lactate could not be evaluated as a marker. In adults, ALT and bilirubin have also been used as markers of graft function [13].

Finally, at one year post-transplantation, children who had a positive fluid balance exceeding 200 mL/kg during the first three postoperative days exhibited higher mean ALT levels compared to those with lower fluid balances. This finding suggests that early postoperative fluid overload may play a role in graft injury and predispose patients to long-term complications, such as liver rejection. However, given the multitude of factors influencing long-term graft function, this association warrants confirmation in future studies. If validated, pediatric liver transplantation protocols should emphasize the maintenance of euvolemia and the avoidance of excessive fluid administration.

This study has several limitations. First, a correlation was demonstrated between lower fluid balance and delta ALT, but, for delta bilirubin, the association was reversed (higher fluid balance with higher delta bilirubin). Moreover, these correlations were only demonstrated in some of the postoperative days, and other markers of fluid overload, such as pulmonary congestion, were not associated with liver function. However, pulmonary congestion is difficult to interpret because pulmonary atelectasis and pleural effusions are very common after pediatric liver transplantation and may mimic congestion on chest radiographs. Possibly, in a larger cohort, correlations between fluid overload and graft injury would be more consistent. Alternatively, fluid overload may lead to liver edema, which manifests as elevated transaminases but not elevated bilirubin.

A second limitation of the study is its retrospective design and the absence of multivariate analysis, which limits the ability to discern whether fluid overload is a cause or result of graft dysfunction and to assess the influence of other contributing factors in graft injury. Fluid overload may contribute to liver edema, potentially increasing resistance to hepatic blood inflow or compressing the inferior vena cava, thereby impairing venous outflow from the graft. Alternatively, patients with graft dysfunction may require more interventions, such as blood product administration, which could account for the higher fluid balance observed. Prospective studies are needed to elucidate this issue.

Third, we excluded two important markers of liver function—lactate and INR—from the analysis. Lactate levels are frequently elevated during adrenaline infusion, which was administered to the majority of our patients, while INR values were influenced by the widespread use of fresh frozen plasma in our cohort. Nevertheless, it is possible that including these parameters might have enhanced the sensitivity of our analysis.

Lastly, fluid balance is only one of many factors affecting graft function following liver transplantation, especially 1 year after the transplantation. One of the most critical factors is graft ischemic time. However, due to the limited availability of ischemic time data in only 16 patients, we were unable to assess its correlation with liver function, which constitutes a significant limitation of our study. Correlations were not found between liver functions and multiple other factors, including baseline disease, donor and graft type, patient age, height, weight, or clinical parameters during transplantation, such as CVP, VIS, and the duration of hypotension episodes.

## 5. Conclusions

In this retrospective, single-center analysis, several correlations were observed between fluid overload and graft injury in pediatric liver transplant patients, although the findings were not consistent. Further studies are needed to better evaluate these associations and to determine whether fluid overload is a cause or a consequence of graft injury. If fluid overload is found to be causal, careful fluid management would be warranted in this specific population.

## Figures and Tables

**Table 1:** Demographic parameters of pediatric liver transplantation patients.

Patients, n.	79
Gender, male, n (%)	43 (54%)
Weight (kg), median (IQR)	15.7 (9.5–22.2)
Height (cm), median (IQR)	98 (73–123)
Age (months), median (IQR)	49 (15–101)
Baseline diagnosis	
Biliary atresia	**20**
Metabolic disorders	**20**
Maple syrup urine disease	3
Wilson disease	3
Hyperoxaluria type 1	2
Propionic academia	4
Methylmalonic academia	2
Ornithine transcarbamylase deficiency	1
Tyrosinemia	1
Alpha-1-antitrypsin	1
Other	3
Cholestasis	**11**
Progressive familial intrahepatic cholestasis	7
Primary sclerosing cholangitis	2
Unknown cause	2
Fulminant hepatic failure	**11**
Hepatoblastoma	**7**
Autoimmune hepatitis	**2**
Budd-Chiari syndrome	**2**
Congenital hepatic fibrosis	**1**
Autosomal recessive polycystic kidney disease	**1**
Alagille syndrome	**1**
Chronic granulomatous disease	**1**
Unknown	**2**

**Table 2:** Clinical characteristics of pediatric liver transplantation patients.

PICU Length of Stay (Days), Median (IQR)	7 (6–13)
Length of mechanical ventilation (days), median (range)	
Invasive	1 (1–17)
Noninvasive	0 (0–30)
Complications during PICU hospitalization, n (%)	
Infections	**33 (42)**
Peritonitis	10
Pneumonia	5
Bacterial	3
Viral	2
Ascending cholangitis	2
Bacteremia	5
Urinary tract infection	2
Clostridium difficile	2
Wound infection	1
Meningitis	1
Vascular complications	**9 (11)**
Hepatic artery thrombosis	4 (8)
Hepatic vein stenosis	4 (8)
Portal vein thrombosis	1 (2)
Biliary leak	**9 (11)**
Bleeding	**8 (10)**
Peritoneal	7
Gastrointestinal	1
Tacrolimus toxicity	**2 (2.5)**

PICU—pediatric intensive care unit.

## Data Availability

The data presented in this study are available on request from the corresponding author due to considerations to patient confidentiality.

## Abbreviations

AKI—acute kidney injury; ALT—Alanine aminotransferase; CVP—Central venous pressure; PICU—Pediatric intensive care unit; POD—Post operative day; SBP—Systolic blood pressure; VIS—Vasoinotropic score.
